# Relationship between sensory characteristics and cortical thickness/volume in autism spectrum disorders

**DOI:** 10.1038/s41398-021-01743-7

**Published:** 2021-12-06

**Authors:** Kaie Habata, Yongjeon Cheong, Taku Kamiya, Daichi Shiotsu, Ichiro M. Omori, Hidehiko Okazawa, Minyoung Jung, Hirotaka Kosaka

**Affiliations:** 1 Department of Neuropsychiatry, University of Fukui, Eiheiji, Fukui, Japan; 2 Cognitive Science Research Group, Korea Brain Research Institute, Daegu, South Korea; 3 Biomedical Imaging Research Center, University of Fukui, Eiheiji, Fukui, Japan; 4 Research Center for Child Mental Development, University of Fukui, Eiheiji, Fukui, Japan; 5 Division of Developmental Higher Brain Functions, Department of Child Development, United Graduate School of Child Development, University of Fukui, Japan, Eiheiji, Fukui, Japan

**Keywords:** Autism spectrum disorders, Human behaviour

## Abstract

Individuals with autism spectrum disorders (ASDs) exhibit atypical sensory characteristics, impaired social skills, deficits in verbal and nonverbal communication, and restricted and repetitive behaviors. The relationship between sensory characteristics and brain morphological changes in ASD remains unclear. In this study, we investigated the association between brain morphological changes and sensory characteristics in individuals with ASD using brain image analysis and a sensory profile test. Forty-three adults with ASD and 84 adults with typical development underwent brain image analysis using FreeSurfer. The brain cortex was divided into 64 regions, and the cortical thickness and volume of the limbic system were calculated. The sensory characteristics of the participants were evaluated using the Adolescent/Adult Sensory Profile (AASP). Correlation analysis was performed for cortical thickness, limbic area volume, and AASP scores. In the ASD group, there was a significant positive correlation between visual sensory sensitivity scores and the right lingual cortical thickness (*r* = 0.500). There were also significant negative correlations between visual sensation avoiding scores and the right lateral orbitofrontal cortical thickness (*r* = −0.513), taste/smell sensation avoiding scores and the right hippocampal volume (*r* = −0.510), and taste/smell sensation avoiding scores and the left hippocampal volume (*r* = −0.540). The study identified associations among the lingual cortical thickness, lateral orbitofrontal cortical thickness, and hippocampal volume and sensory characteristics. These findings suggest that brain morphological changes may trigger sensory symptoms in adults with ASD.

## Introduction

Autism spectrum disorder (ASD) is a neurodevelopmental disorder characterized by impairments in socialization, communication, and range of interests [1]. It often causes functional impairments and social adjustment problems; however, the neurological basis of these core features is unknown.

The Diagnostic and Statistical Manual of Mental Disorders, Fifth Edition, Text Revision (DSM-5-TR) recently added sensory abnormalities as a core symptom to the diagnostic criteria for ASD [1]. This is because sensory abnormalities in ASD have been reported across all ages and intelligence quotient (IQ) levels [2] and affect daily functioning [3] and academic performance [4]. Sensory symptoms of ASD present in a variety of forms, including sensory hypersensitivity, sensory insensitivity, sensory avoiding, and sensory seeking [5].

The Adolescent/Adult Sensory Profile (AASP) is a self-report questionnaire used for assessing behavioral response patterns to sensory stimuli and sensory symptoms in adults [6]. In studies employing the AASP, ASD participants scored higher than typically developing (TD) participants on low registration, sensation seeking, sensory sensitivity and, sensation avoiding, sensory sensitivity [7]. In studies focusing on auditory and tactile modalities, ASD participants had significantly higher sensory scores than TD participants [8, 9].

Donaldson et al. administered the AASP to the parents of ASD children to determine whether they had ASD-related traits and sensory problems [10]. Parents of multiple ASD children had significantly lower scores on sensation seeking and significantly higher scores on low registration, sensory sensitivity, and sensation avoidance than the parents of TD children [10].

Furthermore, these sensory abnormalities are linked to the traditional core features of ASD, such as social impairments [11]. Individuals with ASD exhibited difficulty in discriminating speech and understanding people in noisy environments [12] and tended to perceive external stimuli, such as touch, more significantly than social rewards [13]. Additionally, some individuals with ASD exhibited selective food rejection, leading to picky eating behavior and extreme food preferences, because of hypersensitivity to vision, taste, temperature, and smell [14, 15].

Hilton et al. [16] examined the relationship between sensory reactivity and social impairment severity in a study involving 36 children with ASD and 26 TD children aged 6–10 years. The ASD group showed significant correlations between sensory system scores and the social scale, with sensory abnormalities in the ASD group being the strongest predictors of social impairment [17].

In addition to behavioral studies, little is known about the neurobiological mechanism of sensory abnormalities in ASD. Brain imaging research on the neurological basis of sensory abnormalities has recently received attention. Several functional brain imaging studies have demonstrated functional connections between sensory processing and specific brain regions. Bonny et al. [18] revealed that the orbitofrontal and insular cortex are involved in the integration of olfaction and gustation in eating behavior in TD participants. Yoshimura et al. administered the AASP to 51 individuals with TD and examined its relationship with the gray matter volume of each brain region obtained from magnetic resonance imaging (MRI) [19]. However, there are only a few brain morphology imaging studies on sensory processing in ASD. Result of functional MRI study suggest that ASDs with sensory hypersensitivity showed abnormal functional connectivity between amygdala and orbitofrontal cortex [20]. The results indicated that the higher the scores for each mode of sensation, the larger the volume of primary and secondary sensory areas [19].

Most ASD studies using brain morphology imaging focused on social deficits, and only a few studies investigated the relationship of brain morphology to sensory symptoms. Thomas et al. reported that social and communication scores on the Autism Diagnostic Interview-Revised correlate with the cortical thickness of the regions involved in social and communication functions, such as the anterior cingulate cortex [21]. Longitudinal studies have also reported that the thinning of the temporal lobe cortex is associated with impaired social skills [22]. Diffusion tensor imaging studies reported that people with ASD experiencing sensory problems and sensory processing disorders have disrupted white matter microstructure. One such study by Owen et al. showed a strong correlation between the posterior white matter, including the posterior corpus callosum, posterior radius, and posterior thalamic radius, and the auditory, multisensory, and inattention scores [23]. However, to the best of our knowledge, no study has examined the relationship between brain morphological features, such as cortical thickness and volume, and sensory symptoms in ASD. Here, we used the AASP to measure the sensory characteristics and evaluate their relation to the brain morphology of adults with ASD. The findings of this study will help to elucidate the neurological basis of sensory abnormalities in ASD and to make a valid ASD diagnosis in difficult-to-diagnose cases.

## Materials/Subjects and Methods

### Participants

Forty-three adults with ASD (29 males, 14 females; mean age = 27.7 ± 5.6, 18–41 years) and 84 TD adults (44 males, 40 females; mean age = 28.3 ± 8.0, 19–53 years) were included in the study. Experienced clinicians (H.K. and I.O.) made the clinical diagnosis of ASD using the DSM-5 (Diagnostic and Statistical Manual of Mental Disorders, Fifth Edition). Exclusion criteria for this study were: (1) history of brain injury, major physical illness, and head trauma (2) intelligence quotients (F-IQs) below 80 (assessed using the Wechsler Adult Intelligence Scale 3rd edition), (3) excessive alcohol consumption, or problems with medication. Several individuals with ASD had comorbid disease, 10 had a history of mood disorders, 2 had obsessive–compulsive disorder, 2 had eating disorders, 1 had panic disorder, and 1 with specific learning disorder. There are no significant differences between comorbid disease with ASD and with ASD in brain morphological changes. Autism characteristics were assessed using the Autism Spectrum Quotient (AQ) and Social Responsiveness Scale (SRS). Sensory characteristics were evaluated using the AASP. The purpose and content of the study were explained to the participants and written consent was obtained. The study was approved by the Ethics Committee of the University of Fukui and conducted in accordance with the ethical standards of the Declaration of Helsinki.

### Neuroimaging procedure

MRI was performed using a 3 Tesla positron emission tomography (PET)/MR scanner (SIGNA PET/MR; General Electric Medical Systems, Milwaukee, WI, USA) with an 8-channel head coil owned by the University of Fukui Hospital. High-resolution T1-weighted anatomical MRI data (repetition time = 6.38 ms, echo time = 1.99 ms, flip angle = 11°, field of view = 256 mm, matrix = 256 256, number of slices = 172, voxel dimension = 1.0 × 1.0 × 1.0 mm) were obtained.

The T1-weighted images were processed using FreeSurfer v5.1.0 (http://surfer.nmr.mgh.harvard.edu/), a highly validated open access software package [24]. This software package automates a few of the procedures and allows quantitative assessments of brain anatomy, such as subcortical volume and cortical morphology measurements, with accuracy comparable to manual methods [25, 26]

Similar to our previous study [27], the anatomical data were preprocessed using the following procedure: Motion and heterogeneity correction, automatic Talairach transformation, intensity normalization, skull strips and subcortical white and gray matter segmentation, white and gray matter tessellation, surface smoothing, and inflation, topology correction, and parcellation. In cortical reconstructions, the quality was checked and corrected by trained professionals. Cortical volumes were analyzed over brain regions using the Desikan–Killiany atlas template [28]. For better understating differences of brain between ASD and TD, whole brain information is described in Fig. S2-S4. The results of motion artifact correction and automatic segmentation were evaluated for data quality by a trained expert. Cortical thickness was also examined in a similar manner.

### Statistical analyses

To investigate the relationship between brain morphological changes and sensory symptoms in ASD, partial correlation analysis was performed among the 24 AASP scores (six modality scores × four quadrant scores), 64 brain cortical regions, and 14 brain volume regions. To avoid potential confounding effects, we included age, full-scale IQ, and sex as covariates in the model. Statistical significance was set at *p* < 0.001. A Fisher r-to-z value transformation was performed to compare the correlations between the ASD and TD groups. All statistical analyses were performed using SPSS version 20 (IBM Corp., Armonk, NY, USA) and VassarStats (http://vassarstats.net/) [29].

## Results and discussion

### Behavioral data

The demographic data of the participants are shown in Table 1. There were no differences in the age, sex ratio, or full-scale IQ score between the ASD and TD groups, while the ASD group had significantly higher AQ and SRS scores than the TD group (Table 1).

**Table 1:** Demographic data of participants.

	TD^1^	ASD^2^	*p*
Subjects (*n*)	84	43	–
Age (years)	28.3 (8.0)	27.7 (5.6)	0.668
range	19–53	18–41	–
Sex (*n*, male/female)	44/40	29/14	0.104
Full Scale IQ^3^	109.6 (11.7)	109.4 (11.7)	0.914
Verbal IQ	109.5 (12.8)	112.0 (13.4)	0.298
Performance IQ	108.0 (12.1)	104.0 (12.9)	0.091
AQ^4^(total)	15.9 (6.1)	32.0 (5.4)	<0.001
SRS^5^(total)	49.3 (18.4)	110.5 (27.2)	<0.001

^1^*TD* typically developing.

^2^*ASD* autism spectrum disorder.

^*3*^*IQ* intelligence quotient.

^4^*AQ* Autism-Spectrum Quotient.

^5^*SRS* Social Responsiveness Scale.

In AASP assessment (Table 2), the ASD group had significantly higher total scores in low registration, sensory sensitivity, and sensation avoiding than the TD group. The ASD group had significantly higher scores than the TD group in the following items: sensation avoiding in taste/smell; low registration and sensory sensitivity in movement; low registration, sensory sensitivity, and sensation avoiding in vision; low registration, sensory sensitivity, and sensation avoiding in touch; low registration and sensation avoiding in activity level; and low registration, sensory sensitivity, and sensation avoiding in auditory (Table 2). The TD group had significantly higher total scores than the ASD group in sensation seeking (Table 2). The TD group also scored significantly higher than the ASD group in sensation seeking in movement and visual sensation seeking (Table 2). Additionally, female with ASD had significantly higher scores in taste, movement, activity level, and low registration (see Fig. S1). For further analyses, sex was entered as a covariate in the model.

**Table 2:** Sensory profile data of participants.

		TD^1^	ASD^2^	*p*
Taste/smell	Low registration	2.90 (1.04)	3.51 (1.56)	0.010
	Sensation seeking	6.94 (2.12)	6.00 (2.18)	0.021
	Sensory sensitivity	2.39 (1.31)	2.74 (1.53)	0.179
	Sensation avoiding	4.43 (1.81)	5.53 (2.21)	<0.001
Movement	Low registration	3.23 (1.15)	4.35 (2.09)	<0.001
	Sensation seeking	7.00 (2.48)	4.12 (2.06)	<0.001
	Sensory sensitivity	5.79 (2.11)	7.28 (2.64)	<0.001
	Sensation avoiding	1.25 (0.67)	1.28 (0.73)	0.824
Visual	Low registration	3.51 (1.37)	4.53 (1.71)	<0.001
	Sensation seeking	5.52 (1.71)	3.86 (1.81)	<0.001
	Sensory sensitivity	6.82 (2.39)	8.26 (3.09)	<0.001
	Sensation avoiding	7.14 (2.15)	8.84 (3.23)	<0.001
Touch	Low registration	4.65 (1.79)	6.26 (2.67)	<0.001
	Sensation seeking	7.57 (2.15)	7.02 (2.17)	0.178
	Sensory sensitivity	9.01 (2.82)	11.47 (3.72)	<0.001
	Sensation avoiding	7.26 (2.66)	9.16 (3.04)	<0.001
Activity level	Low registration	6.61 (2.40)	9.93 (2.69)	<0.001
	Sensation seeking	7.62 (1.89)	7.09 (2.34)	0.175
	Sensory sensitivity	3.10 (1.13)	3.47 (1.32)	0.101
	Sensation avoiding	8.43 (2.19)	11.33 (2.59)	<0.001
Auditory	Low registration	5.70 (1.90)	8.30 (3.05)	<0.001
	Sensation seeking	5.07 (1.91)	4.58 (2.31)	0.205
	Sensory sensitivity	6.35 (2.03)	9.67 (3.18)	<0.001
	Sensation avoiding	5.18 (2.22)	8.47 (3.65)	<0.001
Total score	Low registration	26.61 (6.48)	36.98 (10.21)	<0.001
	Sensation seeking	39.71 (6.93)	32.67 (8.17)	<0.001
	Sensory sensitivity	33.46 (7.27)	42.86 (11.02)	<0.001
	Sensation avoiding	33.69 (7.41)	44.42 (11.50)	<0.001

^1^*TD* typically developing.

^2^*ASD* autism spectrum disorder,

### Correlation analysis between AASP scores & social behavior scores

**Fig. 1:**
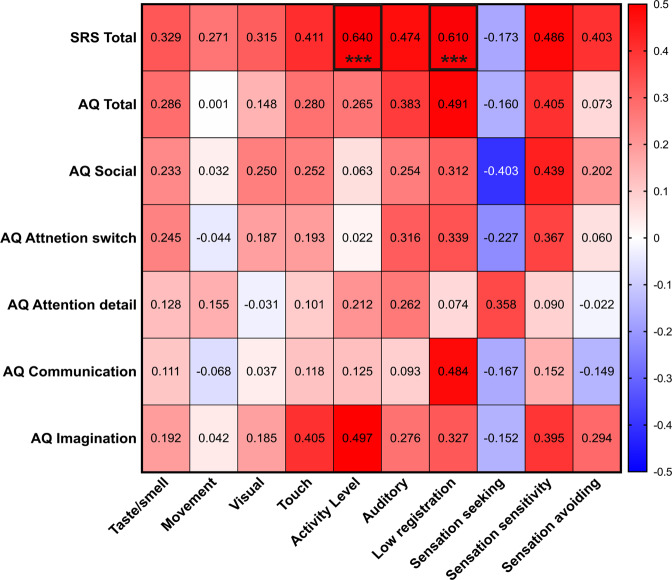
Relationship between sensory profile and social behavior scores in the ASD group. Red colors indicate positive correlations. Bule color indicate negative correlations. Numbers in box indicate correlations coefficients. *ASD* autism spectrum disorder, *AQ* autism spectrum quotient, *SRS* social responsiveness scale. *** *P* ≤ 0.001.

In the ASD group, the correlations corresponding to the SRS total scores were between: activity level (*p* < 0.001*, r* = 0.640) and low registration (*p* < 0.001*, r* = 0.610). Correlation map between AASP score and social behavior scores are shown in Fig. 1 and Table S1.

### Correlation analysis between AASP scores & cortical thickness

**Fig. 2:**
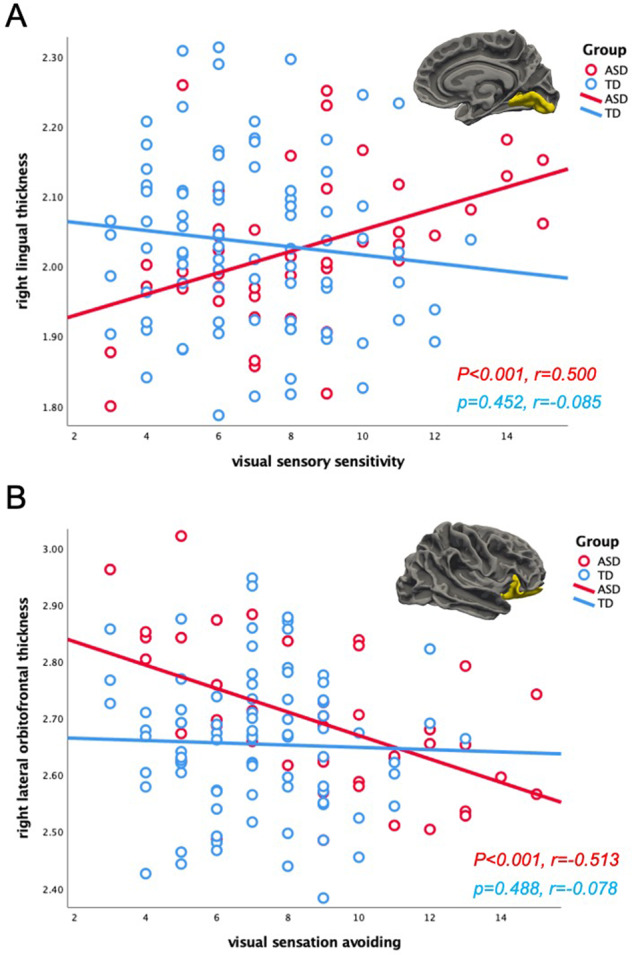
Relationship between AASP scores and cortical thickness. **A** Significant correlation between visual sensory sensitivity scores on the AASP and the right lingual cortical thickness in the ASD group (TD: *p* = 0.452, *r* = 0.085; ASD: *p* < 0.001, *r* = 0.500). The group differences of correlations between the two groups (*z* = 3.28, *p* = 0.001). **B** Significant correlation between visual sensation avoiding scores on the AASP and the right lateral orbitofrontal cortical thickness in the ASD group (TD: *p* = 0.488, *r* = −0.078; ASD: *p* < 0.001, *r* = −0.513). The group differences of correlations between the two groups (*z* = 2.57, *p* = 0.011). *AASP* Adolescent/Adult Sensory Profile, *ASD* autism spectrum disorder, *TD* typically developing.

In the ASD group, there was a significant positive correlation between the visual sensory sensitivity scores on the AASP and the right lingual cortical thickness (*p* < 0.001*, r* = 0.500), while there was a significant negative correlation (*p* < 0.001*, r* = −0.513) between the visual sensation avoiding scores on the AASP and the right lateral orbitofrontal cortical thickness (Fig. 2). No other significant correlations were observed in the ASD group. In the TD group, there were no significant correlations between the AASP scores and cortical thickness. All cortical thickness information is shown in Figs. S2 and S3.

### Correlation analysis between AASP scores & limbic volume

**Fig. 3:**
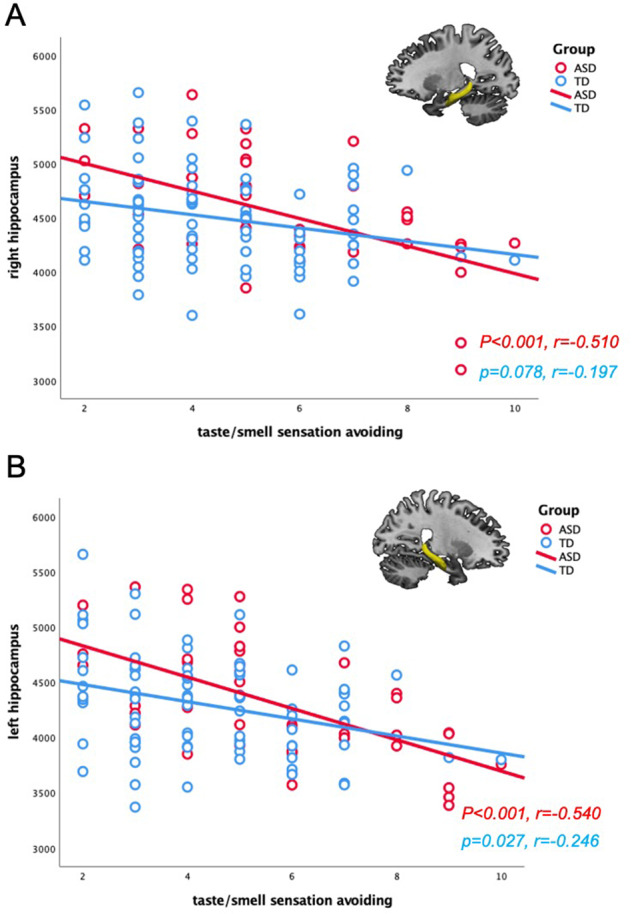
Relationship between AASP scores and limbic volume in the ASD group. **A** Significant correlation between taste/smell sensation avoiding scores and the right hippocampal volume in the ASD group (TD: *p* = 0.078, *r* = −0.197; ASD: *p* < 0.001, *r* = −0.510). **B** Significant correlation between taste/smell sensation avoiding scores and the left hippocampal volume in the ASD group (TD: *p* = 0.027, *r* = −0.246; ASD: *p* < 0.001, *r* = −0.540). *AASP* Adolescent/Adult Sensory Profile, *ASD* autism spectrum disorder, *TD* typically developing.

In the ASD group, significant negative correlations were found between taste/smell sensation avoiding scores on the AASP and the brain volume of the right hippocampus (*p* < 0.001, *r* = −0.510) and left hippocampus (*p* < 0.001*, r* *=* −0.540). No other correlations were detected in the ASD group (Fig. 3). The TD group only showed a significant positive correlation between movement sensation avoiding scores and the right amygdala (*p* < 0.001*, r* = 0.368). All limbic volume information is shown in Fig. S4.

### Correlation differences between the ASD and TD groups

There were group differences between the correlations of the two groups in the visual sensory sensitivity scores and the right lingual cortical thickness (*z* = 3.28, *p* = 0.001). Furthermore, the correlation differences between visual sensation avoiding scores and the right lateral orbitofrontal cortical thickness (*z* = 2.57, *p* = 0.011) were significantly stronger in the ASD group than in the TD group, while the correlation differences between movement sensation avoiding scores and the right amygdala (*z* = 2.64, *p* = 0.001) were significantly stronger in the TD group than in the ASD group.

### Summary

Brain morphology imaging revealed relationships between specific brain regions and sensory characteristics. Particularly, correlations were identified between the visual characteristics and the thickness of the orbitofrontal and lingual cortices as well as between the taste/smell characteristics and the hippocampal volume in adults with ASD. Thus, structural changes in the brains of adults with ASD may cause sensory abnormalities.

### Relationship between sensory behavior and social behavior

Increased sensory abnormality problem was associated with social behavior symptom severity in individuals with ASD. Previous studies have shown that individual with ASD have difficulties in sensory processing, which is reflected social interaction engaged in communication behavior [30–32]. Sensory sensitivity in ASD may also influence performance in social cognition, such as social play, face recognition, and joint attention [33, 34]. Taken together, our results extend these findings by suggesting that sensory processing abnormality may contribute to difficulties in social behavior by disrupting interpersonal communication.

### Lingual cortex

The lingual gyrus is located in the occipital lobe, which contains the primary visual cortex and is central to visual processing. Previous brain morphology imaging studies have reported structural changes in the occipital lobe of patients with ASD [35]. A longitudinal study of ASD showed an increase in cortical thickness and age-related thinning of the visual cortex in childhood ASD [36]. Furthermore, visual hypersensitivity in individuals with ASD was more pronounced in childhood and attenuates in adulthood, suggesting that the intensity of visual hypersensitivity increases with increasing thickness of the visual cortex [35]. This association between cortical thickness and visual characteristics was consistent with the study findings.

A study by Turnbull et al. revealed an association between the autistic traits linked to social interaction and the cortical thickness of the lingual gyrus in ASD [37]. These findings along with the results of the present study suggest that the social impairments in ASD are caused by visual sensory abnormalities.

### Orbitofrontal cortex

Brain structural associations have been reported between the orbitofrontal cortex and the visual characteristics in individuals with ASD. Previous pathological studies have suggested that orbitofrontal cortex dysfunction in ASD affects the limbic system and sensory association cortex, highlighting the association of the orbitofrontal cortex with the cause of social impairments and repetitive and stereotyped behaviors [38]. In a postmortem study of the brain tissue of individuals with ASD, Liu et al. reported alterations in the balance of excitatory and inhibitory neurons in the orbitofrontal cortex [39]. These alterations, which result in abnormal communication between the amygdala and other cortices, including the orbitofrontal cortex, may provide an anatomical basis for the specific features of social interactions and emotions in ASD.

Green et al. using functional brain imaging detected abnormal functional connectivity between the amygdala and orbitofrontal cortex as the neurological basis for sensory symptoms in ASD [20]. Since the orbitofrontal cortex downregulates amygdala activity and regulates responses to sensory stimuli, it is hypothesized that impairments in these functions in individuals with ASD result in hyperactivity to sensory stimuli.

A few researchers have examined the role of structural abnormalities of the orbitofrontal cortex, such as cortical volume and thickness, in ASD. Hardan et al. compared the size of the orbitofrontal cortex in 40 ASD individuals and 41 TD individuals, and it was revealed that the right lateral orbitofrontal cortex was smaller in children and adolescents with ASD, whereas it was larger in adults with ASD [40]. Rooij et al. showed that ASD individuals with higher IQs had a thinner medial orbitofrontal cortex than TD individuals [41].

However, no association between structural abnormalities in the orbitofrontal cortex and sensory symptoms in individuals with ASD has been reported to date. To the best of our knowledge, this is the first study to show an association between the orbitofrontal cortex and sensory symptoms using brain morphological imaging. Dysregulation of networks such as that of the amygdala with the orbitofrontal cortex in ASD is presumed to be due to abnormalities in cortical structures.

In the present study, an association was detected between the orbitofrontal cortex and the visual system. As visual information is transmitted to the visual cortex, it diverges into the ventral and dorsal pathways that have outputs to the hippocampus and amygdala. The amygdala has long been considered the neurological basis for ASD [42]. It is associated with symptoms such as abnormal facial recognition and gaze avoidance [42, 43]. Structural abnormalities in the orbitofrontal cortex may affect amygdala functioning and even lead to face processing deficits.

### Hippocampus

Negative correlations were found between smell/taste sensation avoiding scores and bilateral hippocampal volumes as well as between smell/taste sensation avoiding scores and the volume of the amygdala, although at *p* = 0.004.

Previous studies have reported differences in the amygdala and hippocampal volumes between the ASD and TD groups as well as associations between the volume of these regions and the severity of symptoms, which is an area of interest in ASD [38].

The amygdala and hippocampus play a major role in olfaction. The amygdala is part of the primary olfactory cortex. The adjacent hippocampus is not part of the primary olfactory cortex, but it receives strong afferent input from the entorhinal cortex which is involved in olfactory processing [44]. The role of hippocampal volume changes in Alzheimer’s disease is well known. Furthermore, patients with Alzheimer’s disease exhibit olfactory deficits in proportion to hippocampal atrophy [45]. In a study examining the relationship between olfactory threshold and the amygdala and hippocampal volume in TD participants, a correlation was found wherein as the volume of the right hippocampus increased, the olfactory threshold decreased [46]. However, these results were opposite to the results found in the present study. This may be because ASD pathogenesis differs from that of TD individuals and patients with acquired diseases.

The hippocampus and amygdala are enlarged in adolescents with ASD. Groen et al. suggest that the increase in the amygdala and hippocampal volumes may be due to their excessive activity and use with sensory hypersensitivity [47]. Thus, hippocampal enlargement in adolescents with ASD may be to cope with their inherent hypersensitivity to olfactory sensory. Taken together, our findings demonstrate that lower olfactory threshold was affected by increasing brain volume in olfactory-related regions, such as the amygdala and hippocampus, suggesting that the ASD had an aversion to strong smells and a preference for certain tastes and smells.

There are several limitations in present study. First, we included sex as covariates in the model to control for potential confound gender effects. However, we must consider the possibility that differences in brain morphological changes and sensory symptoms between male and female may have affected the present results. Future study is needed to clarify association between sensory and brain volume in each both males and females with ASD. Second, age range of our sample is very broad, future studies are needed to reveal through adolescence and adulthood. Third, these the AASP questionnaire used in this study was self-administered, which necessitated the inclusion of participants who were linguistically and intellectually high-functioning; thus, the sample size was limited. Although the study group included individuals diagnosed with ASD, they may have milder sensory symptoms; hence, the results may not be generalizable to a wider range of ASD. Thus, it is necessary to increase the sample size and verify the results under strict conditions in which participants with various background factors are included.

In conclusion, we used the AASP, a self-administered sensory processing scoring system, to examine the relationship between sensory characteristics and brain cortical thickness and volume in ASD. These finding have implication for the assessment of abnormal sensory processing in ASD. Significant correlations were found between the thickness of the lingual gyrus and orbitofrontal cortices and visual characteristics as well as between the hippocampal volume and smell/taste characteristics in ASD. These results provide valuable information on brain morphological changes as the neurological basis of sensory abnormalities in ASD.

## Supplementary information

Suplementary

## Data Availability

The data can be made available by request to the corresponding authors.

## References

[1] American Psychiatric Association. DSM-5 Diagnostic Classification. In: *Diagnostic and Statistical Manual of Mental Disorders*. American Psychiatric Association, 2013 10.1176/appi.books.9780890425596.x00diagnosticclassification.

[2] Leekam SR, Nieto C, Libby SJ, Wing L, Gould J (2007). Describing the sensory abnormalities of children and adults with autism. J Autism Dev Disord.

[3] Suarez MA (2012). Sensory processing in children with autism spectrum disorders and impact on functioning. Pediatr Clin North Am.

[4] Howe FEJ, Stagg SD (2016). How sensory experiences affect adolescents with an autistic spectrum condition within the classroom. J Autism Dev Disord.

[5] Baranek GT, David FJ, Poe MD, Stone WL, Watson LR (2006). Sensory experiences questionnaire: discriminating sensory features in young children with autism, developmental delays, and typical development. J Child Psychol Psychiatry Allied Discip.

[6] Catana Brown, Winnie Dunn. *The adolescent/adult sensory profile: user*’*s mannual*. The Psychological Corporation: San Antonio, TX, 2002.

[7] Jao Keehn RJ, Sanchez SS, Stewart CR, Zhao W, Grenesko-Stevens EL, Keehn B (2017). Impaired downregulation of visual cortex during auditory processing is associated with autism symptomatology in children and adolescents with autism spectrum disorder. Autism Res.

[8] Kuiper MWM, Verhoeven EWM, Geurts HM (2019). Stop Making Noise! Auditory sensitivity in adults with an autism spectrum disorder diagnosis: physiological habituation and subjective detection thresholds. J Autism Dev Disord.

[9] Fukuyama H, Kumagaya SI, Asada K, Ayaya S, Kato M (2017). Autonomic versus perceptual accounts for tactile hypersensitivity in autism spectrum disorder. Sci Rep..

[10] Donaldson CK, Stauder JEA, Donkers FCL (2017). Increased sensory processing atypicalities in parents of multiplex ASD families versus typically developing and simplex ASD families. J Autism Dev Disord.

[11] Wigham S, Rodgers J, South M, McConachie H, Freeston M (2015). The interplay between sensory processing abnormalities, intolerance of uncertainty, anxiety and restricted and repetitive behaviours in autism spectrum disorder. J Autism Dev Disord.

[12] Dunlop WA, Enticott PG, Rajan R (2016). Speech discrimination difficulties in high-functioning autism spectrum disorder are likely independent of auditory hypersensitivity. Front Hum Neurosci.

[13] Foss-Feig JH, Heacock JL, Cascio CJ (2012). Tactile responsiveness patterns and their association with core features in autism spectrum disorders. Res Autism Spectr Disord.

[14] Simmons DR, Robertson AE, McKay LS, Toal E, McAleer P, Pollick FE (2009). Vision in autism spectrum disorders. Vis Res.

[15] Bennetto L, Kuschner ES, Hyman SL (2007). Olfaction and taste processing in autism. Biol Psychiatry.

[16] Hilton CL, Harper JD, Kueker RH, Lang AR, Abbacchi AM, Todorov A (2010). Sensory responsiveness as a predictor of social severity in children with high functioning autism spectrum disorders. J Autism Dev Disord.

[17] Hilton CL, Harper JD, Kueker RH, Lang AR, Abbacchi AM, Todorov A (2010). Sensory responsiveness as a predictor of social severity in children with high functioning autism spectrum disorders. J Autism Dev Disord.

[18] Bonny JM, Sinding C, Thomas-Danguin T Functional MRI and Sensory Perception of Food. In: *Modern Magnetic Resonance*. Springer International Publishing, 2017, pp 1–20.

[19] Yoshimura S, Sato W, Kochiyama T, Uono S, Sawada R, Kubota Y (2017). Gray matter volumes of early sensory regions are associated with individual differences in sensory processing. Hum Brain Mapp.

[20] Green SA, Hernandez L, Tottenham N, Krasileva K, Bookheimer SY, Dapretto M (2015). Neurobiology of sensory overresponsivity in youth with autism spectrum disorders. JAMA Psychiatry.

[21] Doyle-Thomas KAR, Duerden EG, Taylor MJ, Lerch JP, Soorya LV, Wang AT (2013). Effects of age and symptomatology on cortical thickness in autism spectrum disorders. Res Autism Spectr Disord.

[22] Wallace GL, Eisenberg IW, Robustelli B, Dankner N, Kenworthy L, Giedd JN (2015). Longitudinal cortical development during adolescence and young adulthood in autism spectrum disorder: Increased cortical thinning but comparable surface area changes. J Am Acad Child Adolesc Psychiatry.

[23] Owen JP, Marco EJ, Desai S, Fourie E, Harris J, Hill SS (2013). Abnormal white matter microstructure in children with sensory processing disorders. NeuroImage Clin.

[24] Fischl B (2012). FreeSurfer. Neuroimage.

[25] Fischl B, Salat DH, Busa E, Albert M, Dieterich M, Haselgrove C (2002). Whole brain segmentation: automated labeling of neuroanatomical structures in the human brain. Neuron.

[26] Fischl B, Salat DH, Van Der Kouwe AJW, Makris N, Ségonne F, Quinn BT (2004). Sequence-independent segmentation of magnetic resonance images. Neuroimage.

[27] Jung M, Tu Y, Lang CA, Ortiz A, Park J, Jorgenson K (2019). Decreased structural connectivity and resting-state brain activity in the lateral occipital cortex is associated with social communication deficits in boys with autism spectrum disorder. Neuroimage.

[28] Desikan RS, Ségonne F, Fischl B, Quinn BT, Dickerson BC, Blacker D (2006). An automated labeling system for subdividing the human cerebral cortex on MRI scans into gyral based regions of interest. Neuroimage.

[29] Lowry R Significance of the difference between two correlation coefficients. Vassarstats. net. 2001; http://vassarstats.net/rdiff.html.

[30] Robertson CE, Baron-Cohen S (2017). Sensory perception in autism. Nat Rev Neurosci.

[31] Williams ZJ, Abdelmessih PG, Key AP, Woynaroski TG (2021). Cortical auditory processing of simple stimuli is altered in autism: a meta-analysis of auditory evoked responses. Biol Psychiatry Cogn Neurosci Neuroimaging.

[32] Beker S, Foxe JJ, Molholm S (2018). Ripe for solution: Delayed development of multisensory processing in autism and its remediation. Neurosci Biobehav Rev.

[33] Case-Smith J, Weaver LL, Fristad MA (2015). A systematic review of sensory processing interventions for children with autism spectrum disorders. Autism.

[34] Thye MD, Bednarz HM, Herringshaw AJ, Sartin EB, Kana RK (2018). The impact of atypical sensory processing on social impairments in autism spectrum disorder. Dev Cogn Neurosci.

[35] Hardan AY, Libove RA, Keshavan MS, et al. A preliminary longitudinal MRI study of brain volume and cortical thickness in autism. *Biol Psychiatry* 2009; **i**:320–6. 10.1016/j.biopsych.2009.04.024 PMC2905654 19520362

[36] Zielinski BA, Prigge MBD, Nielsen JA, Froehlich AL, Abildskov TJ, Anderson JS (2014). Longitudinal changes in cortical thickness in autism and typical development. Brain.

[37] Turnbull A, Garfinkel SN, Ho NSP, Critchley HD, Bernhardt BC, Jefferies E (2020). Word up – Experiential and neurocognitive evidence for associations between autistic symptomology and a preference for thinking in the form of words. Cortex.

[38] Amaral DG, Schumann CM, Nordahl CW (2008). Neuroanatomy of autism. Trends Neurosci.

[39] Liu X, Bautista J, Liu E, Zikopoulos B (2020). Imbalance of laminar-specific excitatory and inhibitory circuits of the orbitofrontal cortex in autism. Mol Autism.

[40] Hardan AY, Girgis RR, Lacerda ALT, Yorbik O, Kilpatrick M, Keshavan MS (2006). Magnetic resonance imaging study of the orbitofrontal cortex in autism. J Child Neurol.

[41] Van Rooij D, Anagnostou E, Arango C, Auzias G, Behrmann M, Busatto GF (2018). Cortical and subcortical brain morphometry differences between patients with autism spectrum disorder and healthy individuals across the lifespan: results from the ENIGMA ASD working group. Am J Psychiatry.

[42] Ishitobi M, Kosaka H, Omori M, Matsumura Y, Munesue T, Mizukami K (2011). Differential amygdala response to lower face in patients with autistic spectrum disorders: An fMRI study. Res Autism Spectr Disord.

[43] Golarai G, Grill-Spector K, Reiss AL (2006). Autism and the development of face processing. Clin Neurosci Res.

[44] Sharvit G, Lin E, Vuilleumier P, Corradi-Dell’Acqua C (2020). Does inappropriate behavior hurt or stink? The interplay between neural representations of somatic experiences and moral decisions. Sci Adv.

[45] Marigliano V, Gualdi G, Servello A, Marigliano B, Volpe LD, Fioretti A (2014). Olfactory deficit and hippocampal volume loss for early diagnosis of Alzheimer disease: A pilot study. Alzheimer Dis Assoc Disord.

[46] Smitka M, Puschmann S, Buschhueter D, Gerber JC, Witt M, Honeycutt N (2012). Is there a correlation between hippocampus and amygdala volume and olfactory function in healthy subjects?. Neuroimage.

[47] Groen W, Teluij M, Buitelaar J, Tendolkar I (2010). Amygdala and hippocampus enlargement during adolescence in autism. J Am Acad Child Adolesc Psychiatry.

