# Supplementary material for: Relationship between sensory characteristics and cortical thickness/volume in autism spectrum disorders
Source: Transl Psychiatry. 2021 Dec 6;11:616. doi: 10.1038/s41398-021-01743-7 (PMC8648722; doi:10.1038/s41398-021-01743-7)
Supplement: Supplementary file 1 — Suplementary [file 41398_2021_1743_MOESM1_ESM.docx]

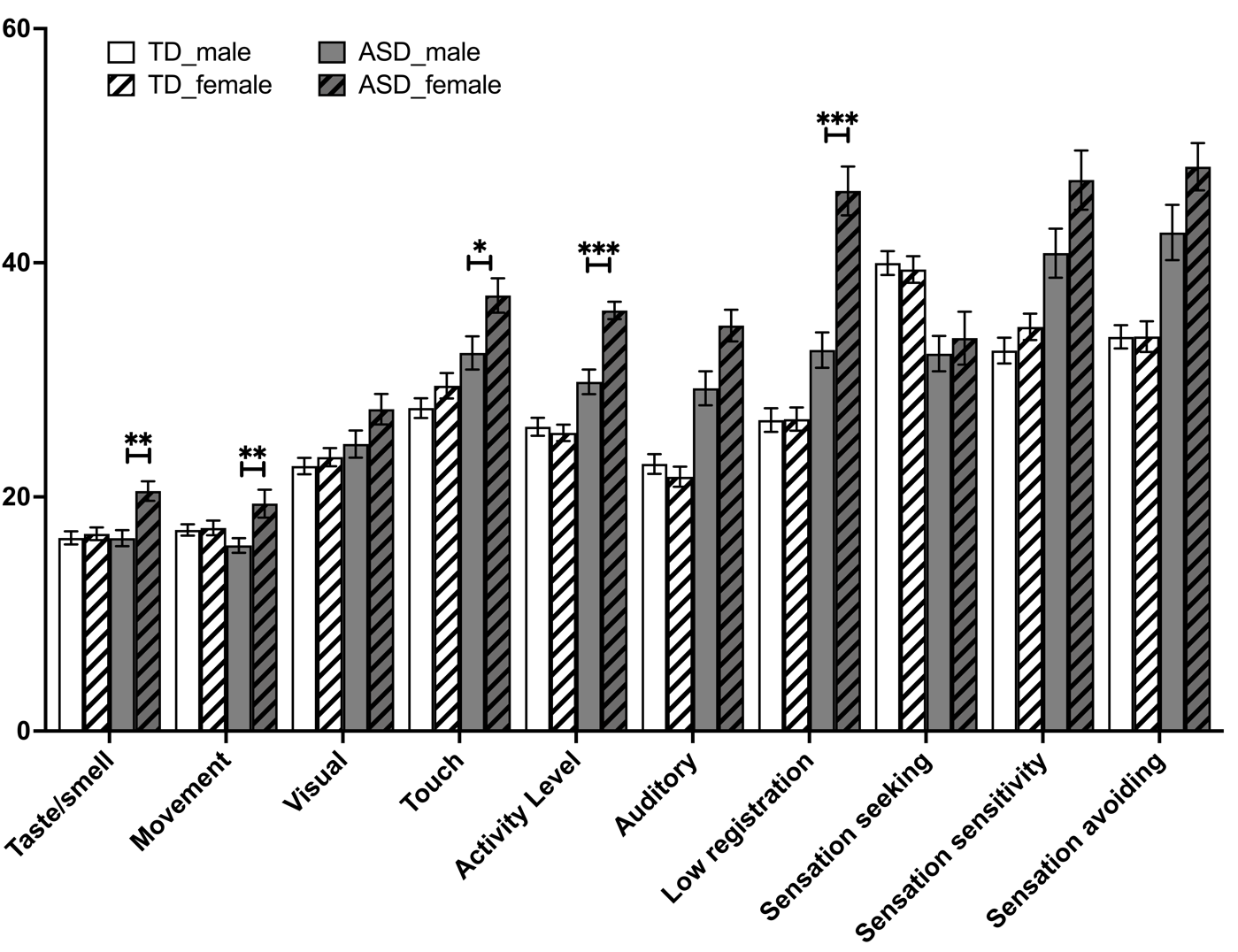


**Figure S1. Sensory profile scores**

Images indicate gender differences of sensory profile scores in each group. Error bars represent standard errors of the mean. **P* ≤ 0.05, ** *P* ≤ 0.01, *** *P* ≤ 0.001


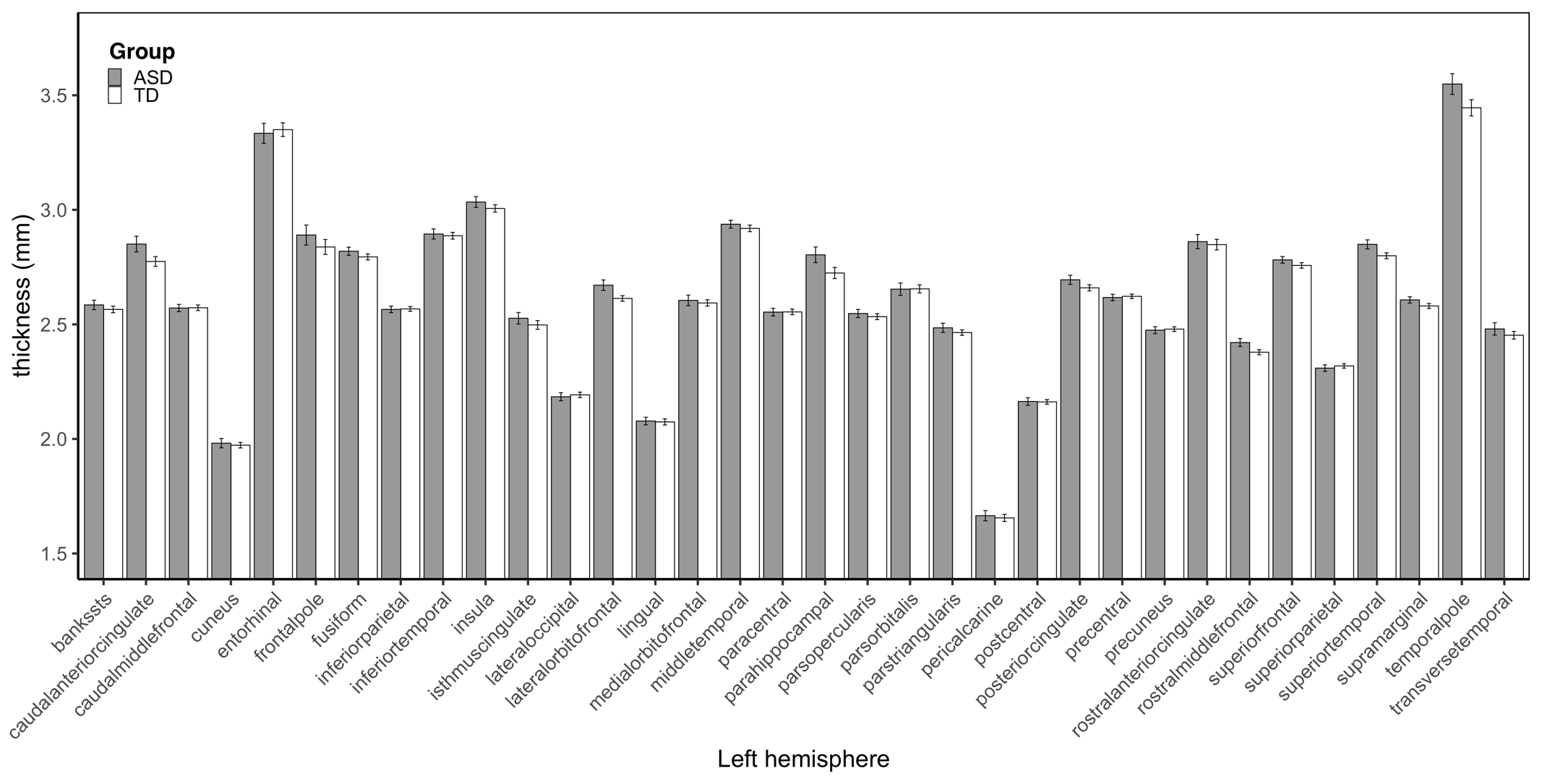


**Figure S2. Left hemisphere cortical thickness information in each group**

Cortical volumes were analyzed over brain regions using the Desikan–Killiany atlas template. Detail information of atlas is described in reference. Desikan, R.S.; Ségonne, F.; Fischl, B.; Quinn, B.T.; Dickerson, B.C.; Blacker, D.; Buckner, R.L.; Dale, A.M.; Maguire, R.P.; Hyman, B.T.; et al. An automated labeling system for subdividing the human cerebral cortex on MRI scans into gyral based regions of interest. *Neuroimage* **2006**, *31*, 968–980, doi:10.1016/j.neuroimage.2006.01.021.


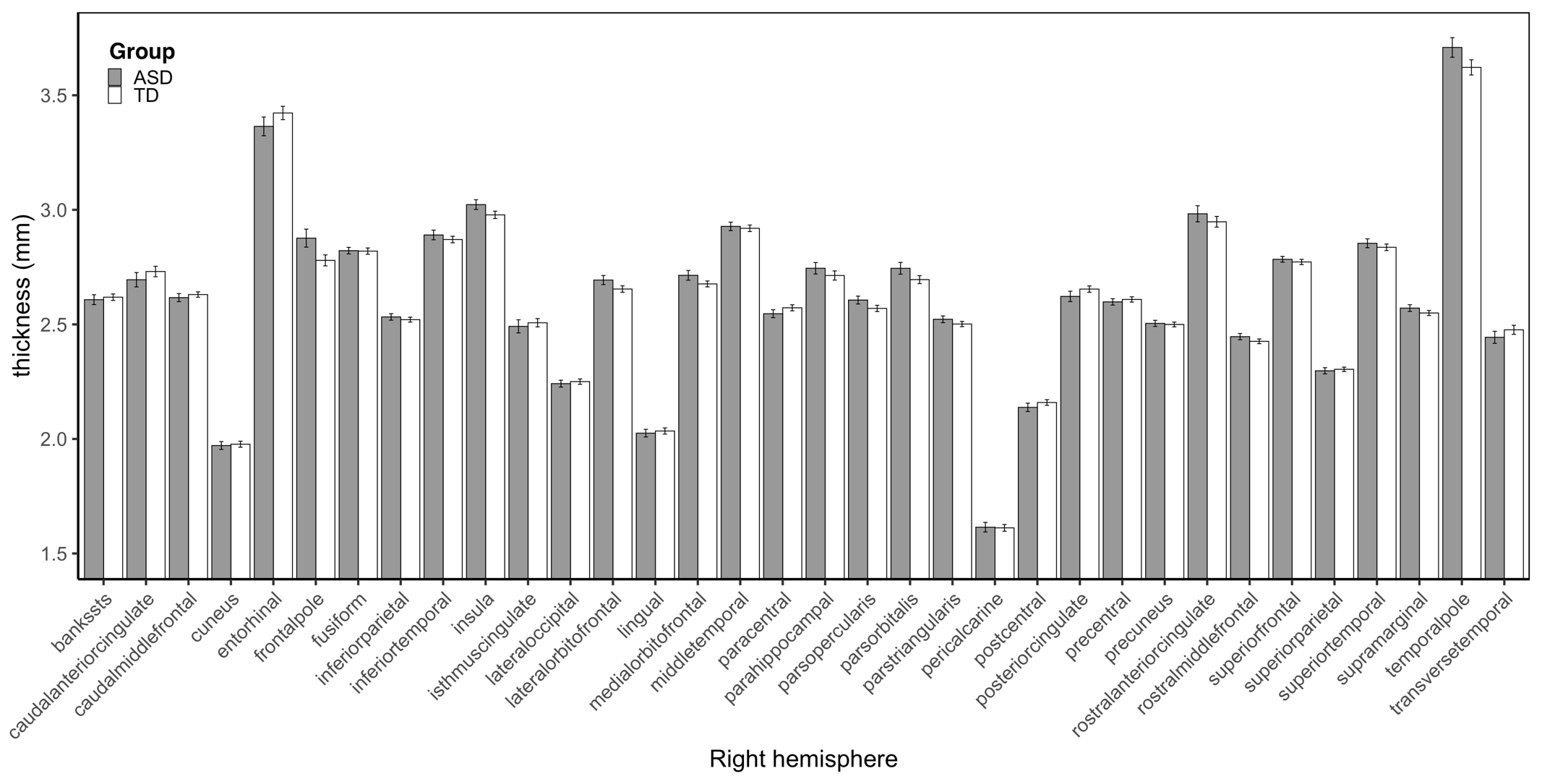


**Figure S3. Right hemisphere cortical thickness information in each group**


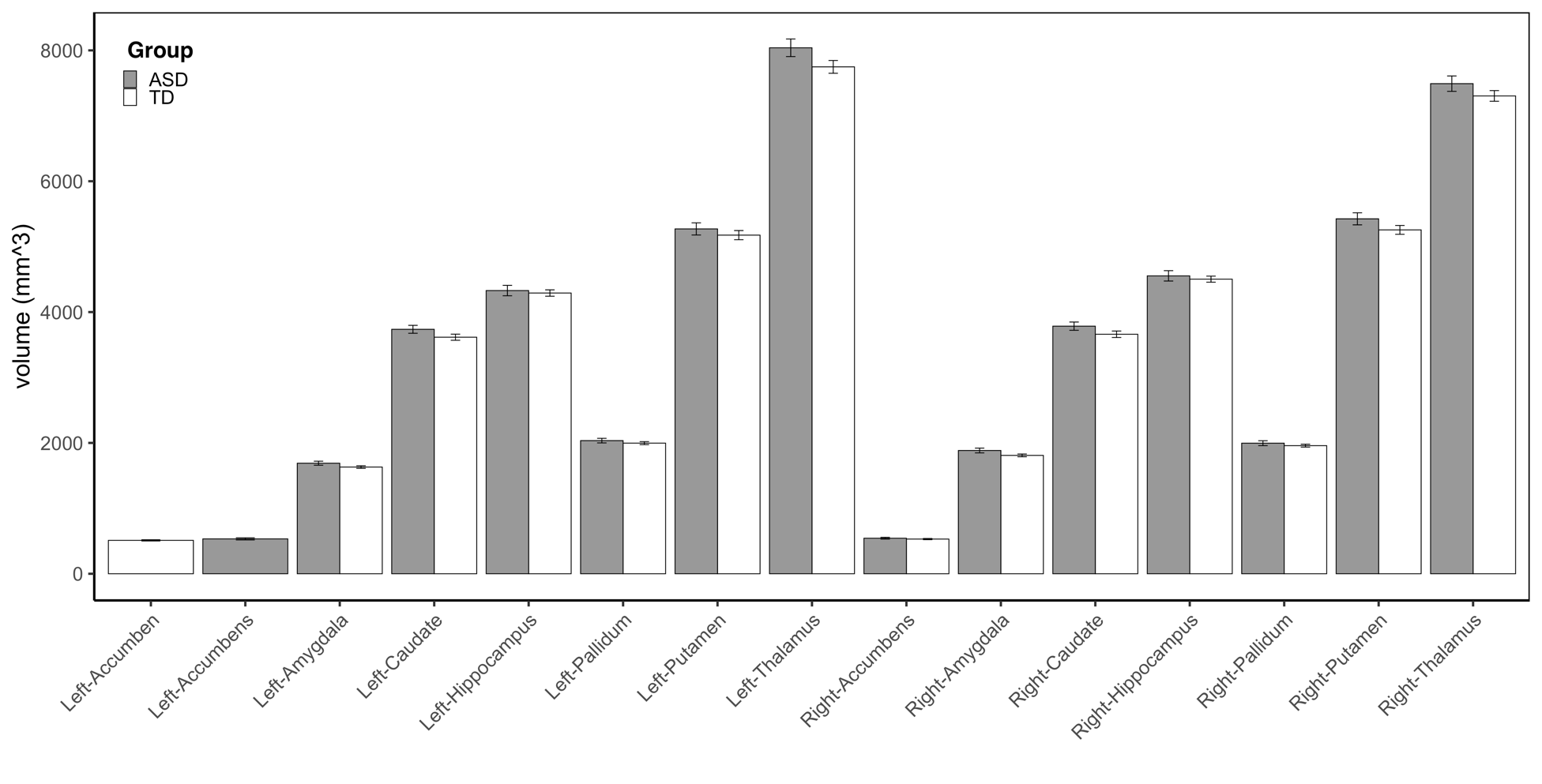


**Figure S4. Limbic volume information**

TableS1. Relationship between sensory profile scores and social behavior socres

|  |  | SRS  Total Score | AQ |  |  |  |  |  |
| --- | --- | --- | --- | --- | --- | --- | --- | --- |
|  |  |  | Total Score | S | AS | AD | C | I |
| Taste / smell | Low registration | 0.192 | 0.259 | 0.231 | 0.217 | 0.089 | 0.253 | -0.161 |
|  | Sensation seeking | -0.026 | -0.008 | -0.209 | -0.15 | 0.291 | 0.051 | -0.019 |
|  | Sensory sensitivity | 0.27 | 0.29 | 0.274 | 0.410** | -0.061 | 0.116 | 0.329* |
|  | Sensation avoiding | 0.258 | 0.101 | 0.26 | 0.128 | -0.116 | -0.134 | 0.231 |
| Movement | Low registration | 0.411** | 0.329* | 0.181 | 0.208 | 0.182 | 0.275 | 0.213 |
|  | Sensation seeking | -0.176 | -0.399* | -0.557** | -0.268 | 0.147 | -0.339* | -0.293 |
|  | Sensory sensitivity | 0.195 | 0.058 | 0.336* | -0.013 | -0.056 | -0.056 | 0.125 |
|  | Sensation avoiding | 0.153 | 0.03 | 0.038 | 0.001 | 0.138 | 0.038 | 0.028 |
| Visual | Low registration | 0.493** | 0.345* | 0.436** | 0.325* | -0.171 | 0.395* | 0.265 |
|  | Sensation seeking | -0.455** | -0.104 | -0.142 | -0.034 | 0.197 | -0.112 | -0.410** |
|  | Sensory sensitivity | 0.289 | 0.258 | 0.367* | 0.25 | -0.051 | 0.09 | 0.309 |
|  | Sensation avoiding | 0.314* | -0.078 | -0.023 | -0.035 | -0.04 | -0.137 | 0.15 |
| Touch | Low registration | 0.184 | 0.132 | 0.176 | 0.154 | -0.226 | 0.23 | 0.134 |
|  | Sensation seeking | -0.015 | -0.081 | -0.162 | -0.065 | 0.138 | -0.097 | -0.039 |
|  | Sensory sensitivity | 0.331* | 0.25 | 0.215 | 0.092 | 0.191 | 0.087 | 0.392* |
|  | Sensation avoiding | 0.403* | 0.288 | 0.292 | 0.255 | 0.074 | 0.055 | 0.369* |
| Activity level | Low registration | 0.573** | 0.408** | 0.05 | 0.211 | 0.22 | 0.359* | 0.416** |
|  | Sensation seeking | -0.011 | -0.11 | -0.371* | -0.297 | 0.315* | -0.188 | 0.131 |
|  | Sensory sensitivity | 0.600** | 0.443** | 0.461** | 0.313* | -0.006 | 0.407** | 0.29 |
|  | Sensation avoiding | 0.448** | 0.031 | 0.2 | -0.028 | -0.076 | -0.108 | 0.329* |
| Auditory | Low registration | 0.553** | 0.433** | 0.265 | 0.226 | 0.147 | 0.377* | 0.296 |
|  | Sensation seeking | -0.066 | 0.032 | -0.148 | -0.059 | 0.281 | 0.001 | -0.06 |
|  | Sensory sensitivity | 0.460** | 0.476** | 0.317* | 0.577** | 0.217 | 0.145 | 0.22 |
|  | Sensation avoiding | 0.146 | -0.018 | 0.112 | -0.02 | 0.033 | -0.238 | 0.16 |
| Total score | Low registration | 0.610** | 0.491** | 0.312 | 0.339* | 0.074 | 0.484** | 0.327* |
|  | Sensation seeking | -0.173 | -0.16 | -0.403* | -0.227 | 0.358* | -0.167 | -0.152 |
|  | Sensory sensitivity | 0.486** | 0.405** | 0.439** | 0.367* | 0.09 | 0.152 | 0.395* |
|  | Sensation avoiding | 0.403* | 0.073 | 0.202 | 0.06 | -0.022 | -0.149 | 0.294 |

AQ autism spectrum quotient, AD attention to detail, AS attention switch, C communication, I imagination, S social, SRS social responsiveness scale. * P ≤ 0.05, ** P ≤ 0.01
